# Predicting Daily Physical Activity in Patients with Chronic Obstructive Pulmonary Disease

**DOI:** 10.1371/journal.pone.0048081

**Published:** 2012-11-02

**Authors:** Arnoldus J. R. van Gestel, Christian F. Clarenbach, Anne C. Stöwhas, Valentina A. Rossi, Noriane A. Sievi, Giovanni Camen, Erich W. Russi, Malcolm Kohler

**Affiliations:** 1 Pulmonary Division, University Hospital of Zurich, Zurich, Switzerland; 2 Department of Health, Zurich University of Applied Sciences, Winterthur, Switzerland; 3 Center for Integrative Human Physiology, University of Zurich, Zurich, Switzerland; University of Giessen Lung Center, Germany

## Abstract

**Background:**

Objectively measuring daily physical activity *(PA)* using an *accelerometer* is a relatively expensive and *time*-*consuming* undertaking. In routine clinical practice it would be useful to estimate PA in patients with chronic obstructive pulmonary disease (COPD) with more simple methods.

**Objectives:**

To evaluate whether PA can be estimated by simple tests commonly used in clinical practice in patients with COPD.

**Methods:**

The average number of steps per day was measured for 7 days with a SenseWear Pro™ accelerometer and used as gold standard for PA. A physical activity level (PAL) of <1.4 was considered very inactive. U*nivariate and multivariate analyses were used to examine the relationship between* the 6-minute walking distance (6MWD), the number of stands in the *Sit-*to-*Stand Test* (STST), hand-grip strength and the total energy expenditure as assessed by the Zutphen Physical Activity Questionnaire (TEE_ZPAQ_). ROC curve analysis was used to identify patients with an extremely inactive lifestyle (PAL<1.4).

**Results:**

In 70 patients with COPD (21 females) with a mean [SD] FEV_1_ of 43.0 [22.0] %predicted, PA was found to be significantly and independently associated with the 6MWD (r = 0.69, 95% CI 0.54 to 0.80, p<0.001), STST (r = 0.51, 95% CI 0.31 to 0.66, p = 0.001) and TEEZPAQ (r = 0.50, 95% CI 0.30 to 0.66, p<0.001) but not with hand-grip strength. However, ROC curve analysis demonstrated that these tests *cannot be used to* reliably identify patients with an extremely inactive lifestyle.

**Conclusions:**

In patients with COPD simple tests such as the 6-Minute Walk Test, the *Sit-*to-*Stand Test* and the Zutphen Physical Activity Questionnaire *cannot be used* to reliably predict physical inactivity.

## Introduction

Physical inactivity in daily life is a prominent feature in patients with chronic obstructive pulmonary disease (COPD) [1]–[4]. Reduced physical fitness may lead to a shift in patients’ lifestyle with low daily physical activity levels (PA) inducing a vicious circle of decreased exercise tolerance, which in turn further reduces activity levels and increases social isolation and depression [5].

The Global Initiative for Chronic Obstructive Lung Disease (GOLD) states that increased participation in physical and social activities of daily living should be among the pertinent clinical *issues in the management of patients with COPD* [6]. Due to the impact of impaired PA on the health status in patients with COPD, accurately estimating the amount and intensity of physical activity in daily life is considered very important [7].

Physical performance in patients with COPD has been assessed mostly by direct observation, by subjective methods such as self-reported questionnaires and diaries [8] and by objective methods such as accelerometers, pedometers [9] and physical fitness tests such as the 6-Minute Walk Test (6MWT), the *Sit-*to-*Stand Test* (STST) [10] and the Hand-grip strength Test [11]. Objectively measuring *PA* using *accelerometry* seems to be the most *accurate* field-based estimate of *PA* [12], however, it is a relatively expensive and *time*-*consuming* undertaking. In routine clinical practice it would be useful to estimate PA with less expensive, less time-consuming and more practical methods. Therefore, the aim of the present study was to investigate if the 6MWT, STST, Hand-grip strength Test and Zutphen Physical Activity Questionnaire (ZPAQ) can accurately predict daily PA in patients with COPD.

### Study Subjects

Patients with COPD referred to the Pulmonary Division, University Hospital of Zurich, Switzerland between January 2010 and August 2011 were considered for participation in the study. The inclusion criteria for patients were: male/female subjects aged 40–75 yrs and confirmed COPD according to GOLD-guidelines. The exclusion criteria were: acute or recent (within last 6 weeks) exacerbation of COPD according to GOLD-guidelines, patients on long-term oral corticosteroids or morphine medication and mental or physical disability precluding informed consent or compliance with the protocol. The study was approved by the Research Ethics Committee of the University Hospital of Zurich, Switzerland (EK-1734) and written informed consent was obtained from all patients.

## Methods

### Pulmonary Function

Spirometry, whole body plethysmography and diffusing capacity measurements were performed according to the American Thoracic Society (ATS) and the European Respiratory Society (ERS) guidelines with a commercially available system [12], [13].

### Daily Physical Activity, Accelerometry

A multisensor accelerometer (SenseWear Pro™ armband; BodyMedia, Inc., Pittsburgh, PA, USA) which is worn on the upper right arm was used. The device estimates energy expenditure (EE) using measurements from a biaxial accelerometer and sensors that quantify galvanic skin response, heat flux and skin temperature. The biaxial accelerometer records the number of steps and the duration of physical activity (PAD) [14]. The physical activity level (PAL) was calculated by dividing the total daily energy expenditure by energy expenditure during sleep [15]. A PAL ≥1.70 defines a moderate to extremely active person, 1.40–1.69 defines a sedentary person, and <1.40 defines an extremely inactive person [15]–[17]. The patients were instructed to wear the accelerometer continuously during 7 consecutive days, except when bathing or showering.

### 6-Minute Walking Test

Patients performed the 6MWT following pulmonary function testing. 6MWT distance was measured according to the guidelines of the American Thoracic Society (ATS) [18]. The 6MWT was performed on a 30-meter indoor track by an experienced investigator using standardized encouragement strategy. None of the p*atients used* a *walking aid in daily life or during* the test.

### Sit-to-Stand Test

A standard chair (height 46 cm) with no arm supports was used. The patients were instructed to stand up from and sit down on the chair with no support from the hands, repeating the procedure as many times as possible for a duration of one minute at a patient-defined pace [10].

### Hand-grip Strength Test

Skeletal muscle strength of the hand was estimated based on handgrip strength of the dominant hand measured with a dynamometer as described elsewhere (Hand-Dynamometer Bremshey; Accell Fitness, Almere, Netherlands) [19].

### Zutphen Physical Activity Questionnaire

The ZPAQ has been used to characterize PA in daily life in patients with COPD [20]. The ZPAQ [21] is a self-reported physical activity questionnaire and addresses the frequency and duration of the patients` activities of the previous week; the average amount of time spent weekly on “homely activities”, “gardening”, “hobbies” and the average amount of time spent monthly on “jobs” and “sports”. According to the frequency, intensity and duration of these activities, a summary Metabolic Equivalent (MET) score expressed in kcal/kg/day is calculated based on an intensity code for each activity, as described by Durrin and Passmore [22] and the Minnesota leisure-time physical activity questionnaire as described by Jacobs [23] and Folsom [24].

### Data Analysis

A statistical software package was used for all calculations (SPSS for Windows, version 11.0, SPSS Inc., Chicago, IL, USA). Descriptive data for continuous variables *are expressed* as *mean and standard deviation*. *Univariate (*Pearson’s correlation) *and multivariate analyses were performed* to evaluate the association between PA (the average number of steps per day) and the 6MWT, STST, Hand-grip Test as well as the ZPAQ. The multivariate analysis included the average number of steps per day derived from accelerometry as the dependent and either 6MWT, STST, Hand-grip strength Test or ZPAQ as well as partial pressure of oxygen (PaO_2_), forced expiratory volume in one second (FEV_1_), age and body mass index (BMI) as independent variables. ROC curve analysis was used to compare the predictive ability of the 6MWT, STST, Hand-grip strength Test and the ZPAQ and to determine the most useful threshold to identify subjects with extremely low physical activity (PAL<1.4). A p-value of <0.05 was considered to indicate statistical significance.

## Results

### Study Profile and Patients’ Characteristics

**Figure 1:**
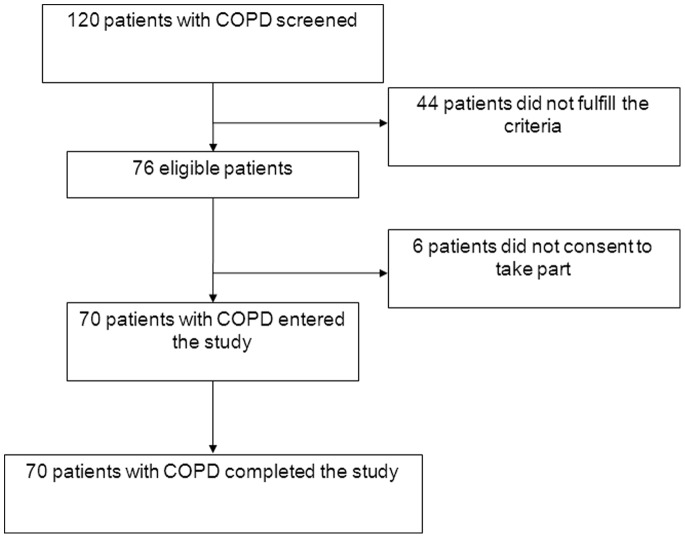
Study profile.

Figure 1 shows the study profile. Seventy patients with COPD agreed to take part and were included in the study. Anthropometrical characteristics and pulmonary function data of the patients are presented in table 1. COPD was mild (GOLD I) in 23.9%, moderate (GOLD II) in 8.5%, severe (GOLD III) in 31.0%, and very severe (GOLD IV) in 36.6% of the patients.

**Table 1:** Anthropometrics and Pulmonary Function.

Variable		
Anthropometrics		
Subjects (n)	70	
Female/male	21/49	
Age (years)	62.4 (7.4)	
BMI (kg/m^2^)	25.0 (7.7)	
Pulmonary function		
FEV_1_ (l)		1.2 (0.7)
FEV_1_ (% predicted)		43.0 (22.0)
FEV_1_/FVC (ratio)		43.6 (15.0)
DLCO (% predicted)		48.2 (23.6)
TLC (% predicted)		116.8 (20.9)
RV/TLC (ratio)		0.57 (0.14)
PaO_2_ (kPa)		9.2 (1.9)
PaCO_2_ (kPa)		5.2 (0.8)

Values are presented as mean (SD). BMI: body mass index; FEV_1_: forced expiratory volume in one second; FEV_1_/FVC ratio: forced expiratory volume in 1 sec (FEV_1_) expressed as percent of FVC; DLCO: diffusion capacity for carbon monoxide; TLC: total lung capacity; RV/TLC: residual volume/total lung capacity ratio; PaO_2_: partial pressure of oxygen; PaCO_2_: partial pressure of carbon dioxide.

### Physical Activity

Data describing physical activity are summarized in table 2. The mean PAL of the patients was 1.47 (0.23); 42.2% of the patients had an extremely inactive lifestyle (PAL<1.4), 40.2% of the patients had a sedentary lifestyle (PAL 1.40–1.69) and 17.6% of the patients were classified as moderate to vigorously active (PAL≥1.70). Mean total energy expenditure (TEE_ACC_) estimated by accelerometry was higher than total energy expenditure (TEE_ZPAQ_) assessed by the self-reported physical activity questionnaire: 2200 [478] and 1292 [1093] kcal/day, respectively (mean difference 922 [95] kcal/day, 95% CI 703 to 1141 kcal/day, p<0.001).

**Table 2:** Physical Performance.

Variable	
Physical fitness	
6MWD (m)	384.3 (136.4)
6MWD (% predicted)	63.4 (21.2)
*Sit-*to-*Stand Test (n)*	20 (6)
Hand-grip Test (kg)	37.3 (10.2)
Daily physical activity by accelerometry	
PAL (ratio)	1.47 (0.23)
MET (kcal/h/kg)	1.2 (0.22)
TEE_ACC_ (kcal/day)	2200 (478)
TSA>3METs (min/day)	55.2 (62.23)
Steps/day (n)	5272 (3319)
Questionnaire-based daily physical activity	
Stairs per week (n)	7.6 (9.4)
Total (MET/week)	118.1 (96.1)
TEE_ZPAQ_ (kcal/day)	1292 (1093)

Values are presented as mean (SD). PAL: physical activity level; MET: metabolic equivalent; TEE_ACC_: total energy expenditure per day as assessed by accelerometry; TSA>3METs: time spend per day in activities demanding more than 3 metabolic equivalents; steps/day: number of steps per day; TEE_ZPAQ_: total energy expenditure per day as assessed by the Zutphen Physical Activity Questionnaire.

### Relationship between Accelerometry and Physical Performance Tests

**Figure 2:**
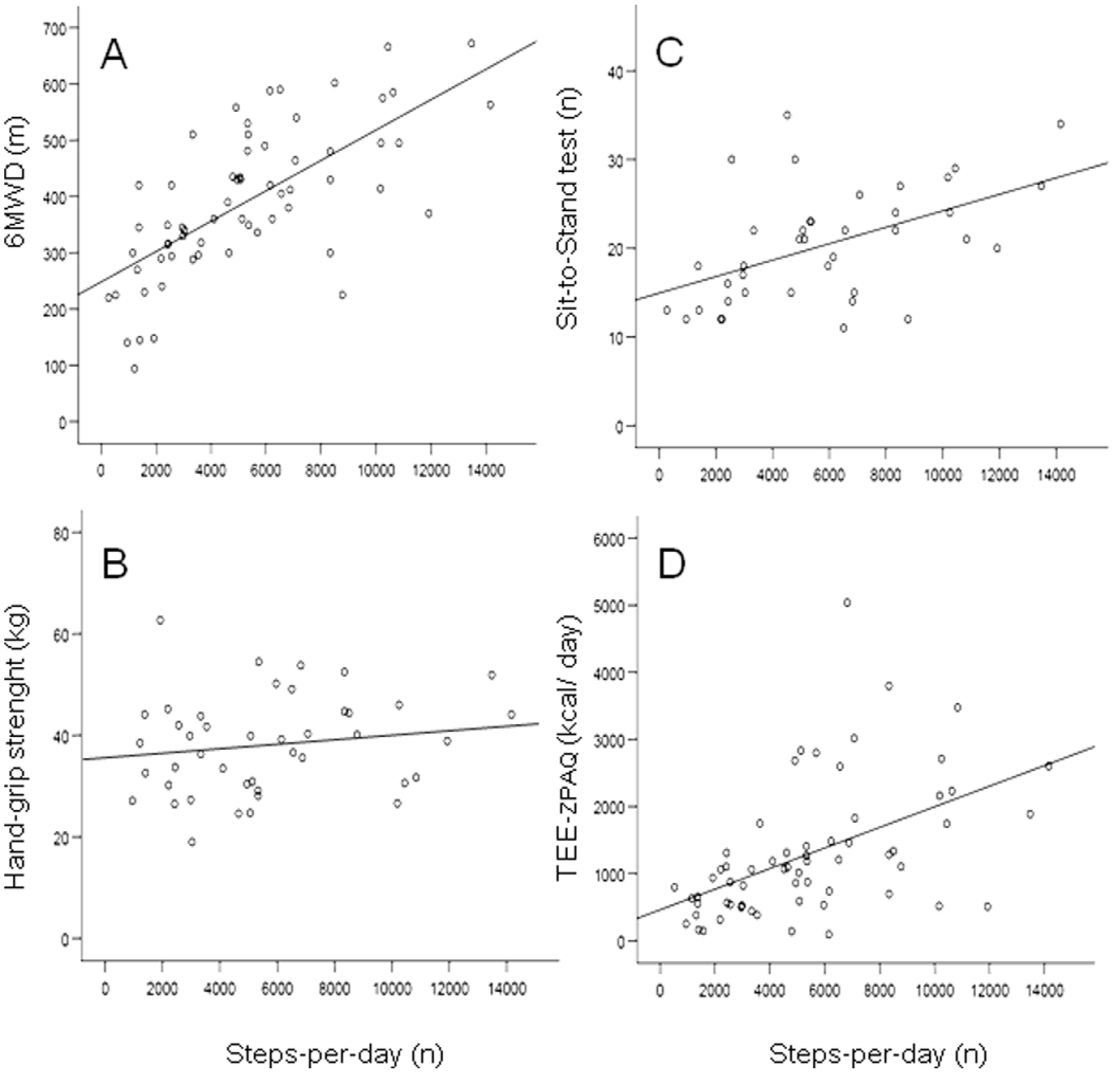
Scatterplots showing the relationship between PA (the average number of steps per day, n) and A: the 6-Minute Walking Distance, 6MWD (r = 0.69, 95% CI 0.54 to 0.80, p<0.001), B: hand-grip strength (r = 0.21, 95% CI −0.03 to 0.42, p = 0.190), C: Sit-to-Stand Test, STST (r = 0.51, 95% CI 0.31 to 0.66, p = 0.001) and D: the total energy expenditure as assessed by the Zutphen Physical Activity Questionnaire, TEE_ZPAQ_ (r = 0.50, 95% CI 0.30 to 0.66, p<0.001).

There was a statistically significant positive correlation between the average number of steps per day measured by accelerometry and the 6-minute walking distance (6MWD), the number of stands during the STST, the metabolic equivalent as measured by accelerometry (MET), the total energy expenditure estimated by accelerometry (TEE_ACC_), the time spent per day on activities demanding more than 3 metabolic equivalents (TSA) and the total energy expenditure as assessed by the self-reported physical activity questionnaire (TEE_ZPAQ_) (table 3, figure 2).

**Table 3:** Correlations with Average Steps per Day.

Variable	Coefficient r	95% CI	p-Value
Performance tests			
6MWD (m)	0.69	0.54–0.80	<0.001
Sit-to-Stand Test (n)	0.51	0.31–0.66	0.001
Hand-grip strength Test (kg)	0.21	−0.03–0.42	0.19
Performance-based dailyphysical activity			
MET (kcal/h/kg)	0.58	0.40–0.72	<0.001
TEE_ACC_ (kcal/day)	0.46	0.25–0.63	<0.001
TSA>3METs (min/day)	0.48	0.28–0.64	<0.001
Questionnaire-based dailyphysical activity			
Stairs per week (n)	0.39	0.17–0.57	0.001
TEE_ZPAQ_ (kcal/day)	0.50	0.30–0.66	<0.001

Correlation is expressed as Pearson’s correlation coefficient. MET: metabolic equivalent; TEE_ACC_: total energy expenditure per day as assessed by accelerometry; TSA>3METs: time spend per day in activities demanding more than 3 metabolic equivalents; TEE_ZPAQ_: total energy expenditure per day as assessed by the Zutphen Physical Activity Questionnaire.

The results of the multivariate analyses are shown in table 4. The average number of steps per day was found to be *independently associated with* the 6MWD, STST and TEE_ZPAQ_. By using 6MWD in the multivariate analysis 49.9% of the *variance in* daily PA could *be explained by* the model (table 4). STST and TEE_ZPAQ_ explained 51.7% and 47.9% of the *variance in PA*, respectively (table 4).

**Table 4:** Multiple Regression Analysis of Predictors of Physical Activity (Steps per Day).

Model 1	CoefficientB	Std.Error	Coefficientβ	t	p-Value
Residual	−2221.65	4172.01		−0.53	0.596
6MWD (m)	14.67	3.40	0.57	4.32	<0.001
PaO_2_ (kPa)	128.29	194.85	0.069	0.66	0.513
FEV_1_(%predicted)	24.13	20.01	0.16	1.21	0.233
Age (years)	8.37	45.37	0.019	0.18	0.854
BMI (kg/m^2^)	−37.83	62.70	−0.063	−0.60	0.549
Model 2					
Residual	817.65	5829.85		0.14	0.889
STST (n)	155.38	73.15	0.28	2.12	0.041
PaO_2_ (kPa)	327.9	307.06	0.16	1.07	0.293
FEV_1_(%predicted)	70.1	25.28	0.41	2.77	0.009
Age (years)	−85.14	61.48	−0.18	−1.39	0.175
BMI (kg/m^2^)	26.05	83.82	0.039	0.31	0.758
Model 3					
Residual	1448.95	3726.93		0.39	0.699
TEE_ZPAQ_(kcal/day)	1.52	0.36	0.46	4.28	<0.001
PaO_2_ (kPa)	243.33	192.16	0.13	1.27	0.211
FEV_1_(%predicted)	59.61	16.28	0.41	3.66	0.001
Age (years)	21.45	46.04	0.05	0.47	0.643
BMI (kg/m^2^)	−161.24	60.38	−0.28	−2.67	0.010

6MWT: 6-Minute Walk Test; STST: *Sit-*to-*Stand Test;* TEE_ZPAQ_: total energy expenditure per day as assessed by the Zutphen Physical Activity Questionnaire; PaO_2_: partial pressure of oxygen; FEV_1_: forced expiratory volume in one second; BMI: body mass index.

### Prediction of Extreme Inactivity

ROC curve analysis revealed that only the 6MWT had modest predictive capacity (area under the curve 0.68). Corresponding analyses for the STST and the ZPAQ showed an area under the curve of 0.31 and 0.43 respectively. Therefore, only the 6MWD was used for further analysis and 425 m appeared as the most useful cut-off point to predict a very inactive lifestyle with a positive and negative predictive value of 0.46 and 0.65 respectively (likelihood ratio of 1.20).

### Relationship between Disease Severity and Physical Performance

**Figure 3:**
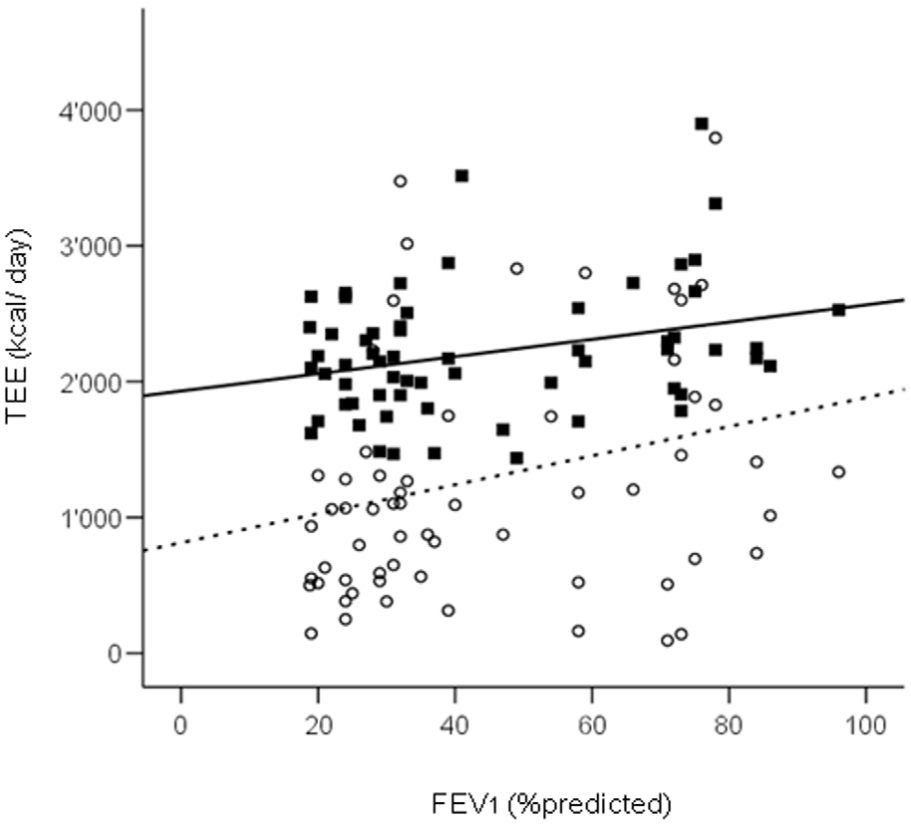
Scatterplots showing the relationship between *FEV_1_ (%predicted)* and the total energy expenditure as assessed by accelerometry, TEE_ACC_ (black squares) (r = 0.32, 95% 0.09 to 0.52, p = 0.011) and by the Zutphen Physical Activity Questionnaire, TEE_ZPAQ_ (white dots) (r = 0.31, 95% CI 0.81 to 0.51, p = 0.012), respectively. Patients with COPD seem to underestimate their daily PA level.

Statistically significant positive *correlations were found between FEV_1_ (%predicted)* and 6MWD *(*r = 0.60, 95% CI 0.43 to 0.73, p<0.001), the average number of steps per day *(*r = 0.50, 95% CI 0.30 to 0.66, p<0.001), STST (n) *(*r = 0.37, 95% CI 0.15 to 0.56, p = 0.014), TEE_ACC_ *(*r = 0.32, 95% CI 0.09 to 0.52, p = 0.011) and TEE_ZPAQ_ *(*r = 0.31, 95% CI 0.28 to 0.44, p = 0.012) (figure 3) but not with hand-grip strength *(*r = 0.059, 95% CI −0.18 to 0.29, p = 0.696).

## Discussion

Methods capable of accurately estimating PA levels and thus quantifying the level of disability are becoming an *increasingly* important *clinical issue in the management of patients with COPD*. Accelerometry is the most accepted method used to measure PA [14]. *We investigated the usefulness of* less expensive, less time-consuming and more practical methods and found that the 6-minute walking distance (6MWD), the number of stands in the STST and the total energy expenditure as assessed by the ZPAQ were independently associated with the average number of steps per day. Despite this, we found that these tests cannot be used to accurately predict an extremely inactive lifestyle in patients with COPD.

In clinical practice, the 6MWT is widely used to assess physical fitness in patients with COPD [25]. The 6MWT is easy to perform, has been standardized [18], and is well tolerated by patients with COPD. Furthermore, the 6MWD has been suggested to be an independent predictor of mortality in patients with COPD [26]. Compared to the age, sex, height and weight matched normal values of Troosters and colleagues [29] the mean 6MWD of our patients was 63.4% predicted. Several authors have proposed that 6MWD and PA as assessed by accelerometry are associated in patients with COPD [9], [27]–[31]. The findings of the current study corroborate these earlier results, however, *ROC curve* analysis *revealed that the 6MWT cannot be used* to reliably predict a very inactive lifestyle.

The STST is an accepted measure of functional status in both elderly people [32] and in patients with COPD [33], [34]. The number of stands during the STST correlates with the BODE Index [26] and is considered a predictor of disease severity [34]. The test is almost self- explanatory and consumes less time, is better tolerated by patients and produces less hemodynamical stress compared to the 6MWT [34]. In the current study, we found that the number of stands during the STST was correlated with FEV_1_ and independently associated with PA. However, *ROC curve* analysis *revealed that* the STST *cannot be used* to predict a very inactive lifestyle.

Pitta and colleagues stated [9] that PA could be better predicted by a “global” or “integrative” test (e.g. 6MWT) rather than by tests focused on single components of physical functioning, such as lung function or muscle strength. In accordance, we found that peripheral muscle strength of the hand as measured by the Hand-grip strength test was not associated with daily PA and thus may not be used to estimate daily physical activity.

The ZPAQ can be self-completed in 15 minutes and showed a good internal reliability, test-retest reliability and validity in a general population of elderly men [24]. In the current study, we found that the daily energy expenditure as assessed by the ZPAQ (TEE_ZPAQ_) was independently associated with PA. This is interesting, as the use of self-reported activity questionnaires has been challenged as a poor measure of actual PA in daily life [35] due to limited validity and reliability [36] as well as due to poor correlation with objectively quantified PA in patients with COPD [37], [38]. It has been speculated that the weak association between self-reported activity and actual PA is due to impaired memory [39], the possibility of misreporting activity time [40] or unnoticed movements [37]. In addition, the effect of social desirability and social approval on self-reports may also influence accurate recall of the type, intensity, frequency and duration of daily PA [41]. Although there was an independent association between TEE_ZPAQ_ and PA, patients with COPD seemed to underestimate their daily PA level (figure 3). On the other hand, this discrepancy can also be explained by the fact that accelerometry overestimates TEE [42]. In addition, we found that the ZPAQ *cannot be used* to identify extremely inactive patients. Thus we conclude that self-reported physical activity needs to be interpreted with care when assessing the activity level of COPD patients.

In this study, we found significant positive *correlations between the severity of COPD (FEV_1_*) and the average number of steps per day, 6MWD, Sit-to-Stand Test, TEE_ACC_ and TEE_ZPAQ_. These findings suggest that with advanced airflow limitation and disease severity, both daily PA and physical fitness are impaired in patients with COPD. In a recently published study by *Garcia*- *Aymerich* and colleagues [43], physical activity of less than 60 minutes per day was considered a risk factor for hospital readmission in patients with COPD. In the current study, we found that 70% of our patients can be considered “at risk”. Furthermore we found that 86% of our patients walked considerably less than the 10,000 steps per day recommended for health promotion [44], [45] and 82.4% of the patients had a sedentary to extremely inactive lifestyle [15]–[17]. These results show that patients with COPD should be actively encouraged to be more active and take part in physical fitness programmes. Interestingly, compared to the PA values, the results of the 6MWT seem to be *more optimistic* in absolute terms. This discrepancy may be due to external factors, such as effort spent, motivation and the instructions and the encouragement given to the subjects participating in a 6MWT in contrast to their usual activity in daily life. In addition, it may be postulated that PAL in patients with COPD are directly linked to depressive syndromes and anxiety [46].

The *present study* has certain *limitations* that need to be taken into account. First, the number of *subjects* is quite small given the variance in data. Second, it is difficult to identify a gold standard measure of daily PA against which to assess the accuracy of different methods. The methods used in this study have different outcomes, with accuracy assessed in various ways. However, the SenseWear Pro™armband used in this study provides a valid and reliable estimate of patients’ average number of steps per day [15] and of energy expenditure [47] during slow and normal walking speed in a laboratory setting [48].

In summary, we found that physical activity in patients with COPD is independently associated with the 6-minute walking distance (6MWD), the number of stands during the STST, the total energy expenditure assessed by the self-reported physical activity questionnaire (TEE_ZPAQ_). These simple tests *cannot be used* to identify patients with an extremely inactive lifestyle.
